# Four-Year Outcomes of Corrective Surgery With Anterior and Posterior Instrumentation Combined With Kyphectomy for a Patient With Congenital Kyphoscoliosis and Underlying Myelomeningocele

**DOI:** 10.7759/cureus.43259

**Published:** 2023-08-10

**Authors:** Lim Hong Ng, Jin Aun Tan, Mohd Hisam Muhamad Ariffin

**Affiliations:** 1 Department of Orthopedics and Traumatology, Faculty of Medicine, Pusat Perubatan Universiti Kebangsaan Malaysia, Kuala Lumpur, MYS; 2 Department of Spine Surgery, Universiti Kebangsaan Malaysia Medical Centre, Kuala Lumpur, MYS

**Keywords:** single-stage surgery, dura, pressure ulcer, gibbus, sitting balance, corrective spine deformity surgery, spinal instrumentation, kyphectomy, myelomeningocele, severe congenital kyphoscoliosis

## Abstract

Patients with myelomeningocele associated with severe kyphoscoliosis usually presented with rigid and angulated gibbus at their back. The condition causes this group of patients to face difficulties in their daily activities, especially in sitting and lying in supine positions. They are also prone to have a pressure sore over the gibbus and encounter the risk of infection. Here the authors would present a case of a four-year-old girl with underlying myelomeningocele who was diagnosed with worsening kyphoscoliosis along her growth. Her whole spine x-ray radiograph revealed a kyphosis angle of 80° between the T11 and L4 levels. The patient underwent a deformity corrective surgery with total kyphectomy in a combination of anterior and posterior spinal instrumentation. In the present case, we were able to obtain sufficient correction of the spinal kyphotic deformity in that patient in a single-stage surgery with satisfactory surgical outcomes at a four years follow-up.

## Introduction

Spinal deformities are common associations seen in patients with myelomeningocele. An estimated 10-15% of newborns with myelomeningocele will present with kyphotic deformity of the spine [1]. The progressive kyphotic deformity causes various disorders including restrictive lung disease, trunk imbalance, significant functional disability, disturbance of the bladder, and bowel dysfunction [2,3]. This kyphosis and its concomitant compensatory thoracic and lumbar lordosis create a bony prominence (gibbus); refractory ulceration of the overlying skin is common.

Conservative treatment of kyphosis with bracing is ineffective in long-term management since kyphosis progresses rapidly with skeletal growth. High-level myelomeningocele patients may also develop progressive scoliosis with the incidence and severity of scoliosis directly correlating to the level of motor dysfunction.

Therefore, surgical treatments were performed during infantile, juvenile, or adolescent periods in the majority of cases [4]. Kyphectomy is the surgery of choice in cases of rigid myelomeningocele-related kyphosis but is associated with numerous complications, including skin and wound infection, non-fusion, and others.

## Case presentation

**Figure 1:**
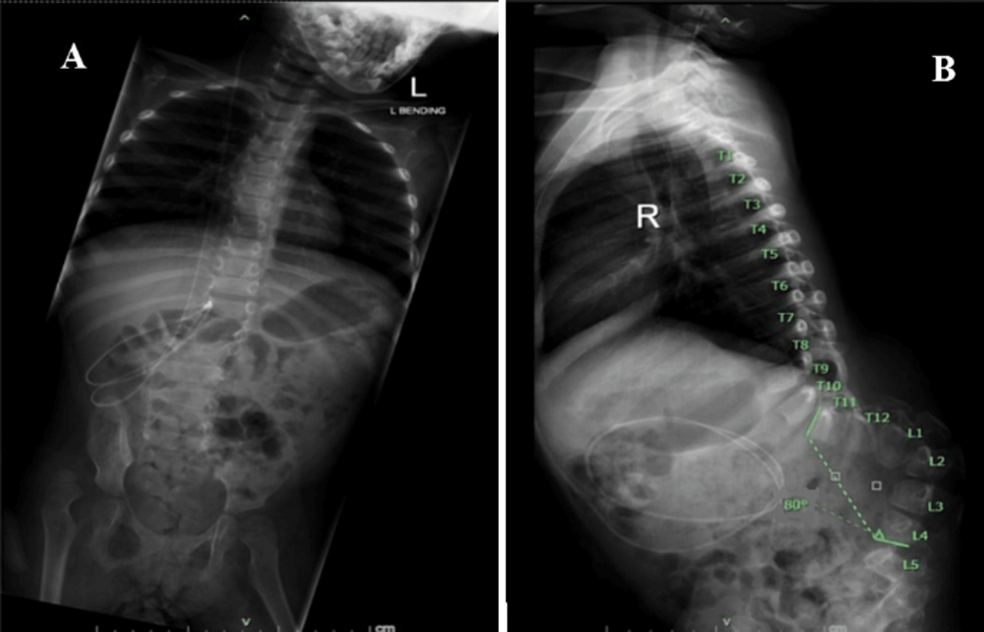
Pre-operative whole spine x-ray. Anteroposterior view (A) and lateral view (B) show kyphosis of 80° between T11 and L4 levels.

A four-year-old girl with underlying myelomeningocele presented to us with worsening kyphoscoliosis curvature and recurrent ulceration at the apex of the kyphosis along her growth. A whole spine x-ray radiograph revealed a kyphosis angle of 80° between the T11 and L4 spinal levels (Figure 1, panels A and B). The patient underwent a single-stage deformity corrective surgery of total kyphectomy in combination with anterior and posterior spinal instrumentation.

**Figure 2:**
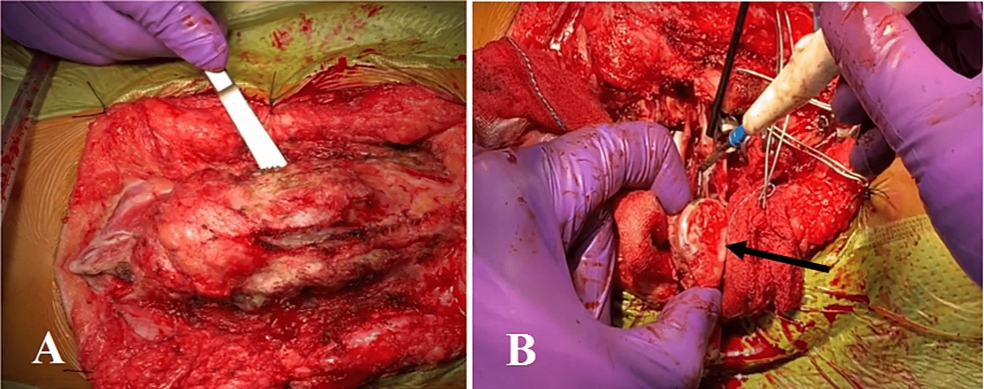
Thick fibrous tissue was noted adhering to the undersurface of the dura. Thick fibrous tissue was dissected, released, and transected at the level of defect (A). A total kyphectomy of the L3 vertebrae (arrow) was performed (B).

Surgery was done under general anesthesia, and the patient was continuously monitored using neuro-electrophysiological monitoring. The patient was positioned in a prone position on the operation table. The level of the vertebra was identified under image intensifier guidance. A mid-line incision was made and the surgical wound was opened in layers until the posterior elements of the spine were exposed. Intra-operatively, thick fibrous tissue was noted adhering to the undersurface of the dura. It was dissected, released, and transected at the level of defect followed by total kyphectomy of the L3 vertebrae (Figure 2, panels A and B, and Video 1).

**Video 1:** Summarized video for the surgery of the patient. Intra-operatively, thick fibrous tissue was noted adhering to the undersurface of the dura. It was dissected, released, and transected at the level of defect followed by total kyphectomy of the L3 vertebrae. Then anterior instrumentation was performed from L1 to L5 level in combination with posterior instrumentation from T10 level to pelvis.

**Figure 3:**
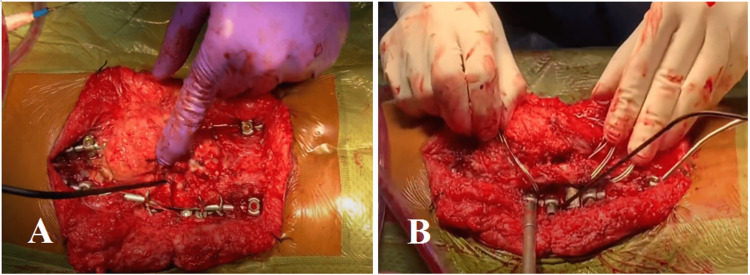
Anterior instrumentation was performed from L1 to L5 levels. Anterior instrumentation was performed in combination with posterior instrumentation from T10 to pelvis (A). The kyphoscoliotic curvature of the spine was corrected and stabilized with one short rod and two long rods augmented with translaminar wire cerclage (B).

**Figure 4:**
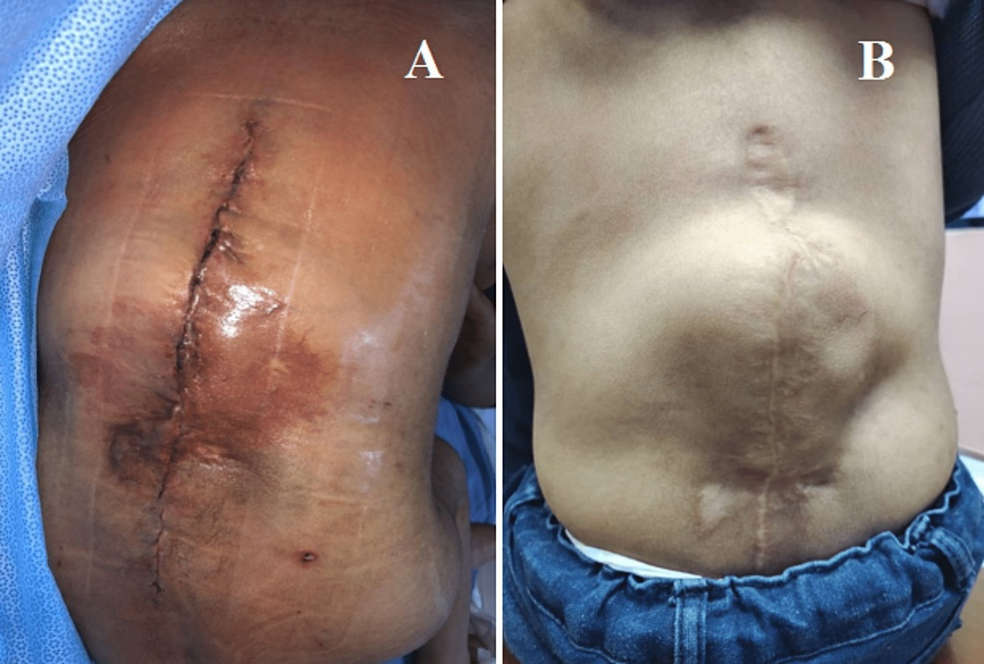
Skin closure was completed with subarticular suturing method using Monosyn 2/0 after the surgery (A). The surgical site healed well with no recurrent ulceration at a four years follow-up in the clinic (B).

Then anterior instrumentation was performed from L1 to L5 in combination with posterior instrumentation from T10 to the pelvis (Figure 3, panels A and B, and Video 1). The kyphoscoliotic curvature of the spine was corrected and stabilized with one short rod and two long rods augmented with translaminar cerclage wire (Figure 3, panels A and B, and Video 1). Distal control of the constructs was achieved with the iliac screw together with pedicle screws and sublaminar wire. Skin closure was completed with subarticular suturing method using Monosyn 2/0 after the surgery (Figure 4, panel A).

One spine surgeon and one neurosurgeon performed simultaneous exposure of the spine, insertion of pedicle screws, and kyphectomy. Operating time was 3 hours and 30 minutes and the patient was hemodynamically stable intra-operatively. Post-operative spine radiograph under an image intensifier showed good screw placement and acceptable spinal alignment. The patient was sent back to the ward post-operatively for monitoring.

**Figure 5:**
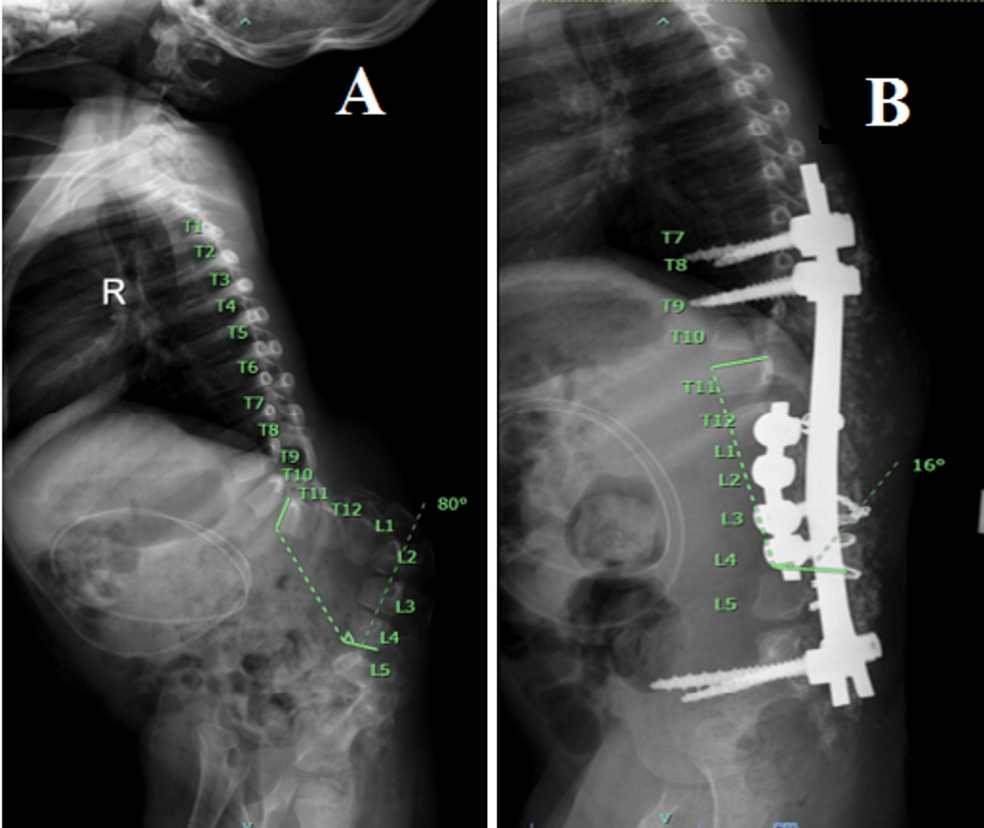
The pre-operative kyphosis of 80° (A) was corrected to 16° post-operatively with improved sitting posture (B).

**Figure 6:**
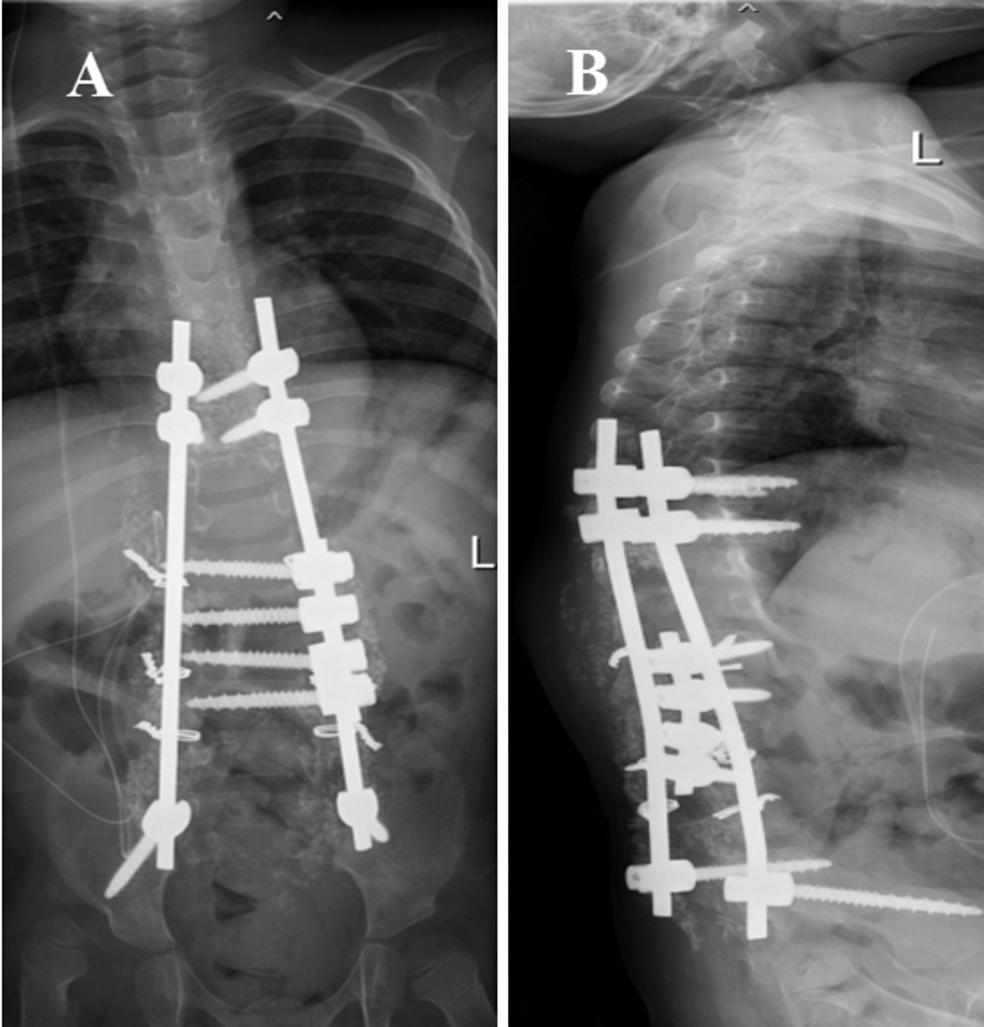
Post-operative whole spine x-ray. The images, anteroposterior view (A) and lateral view (B), show acceptable correction of thoracolumbar kyphosis with a combination of anterior and posterior instrumentation.

Post-operatively, the patient showed stable vital signs and good pain control under acute pain service team co-management. The pre-operative kyphosis of 80° was corrected to 16° post-operatively (Figure 5, panels A and B). In the present case, we were able to obtain satisfactory correction of the spinal kyphotic deformity in a single-stage surgery with a satisfactory outcome (Figure 6, panels A and B).

**Figure 7:**
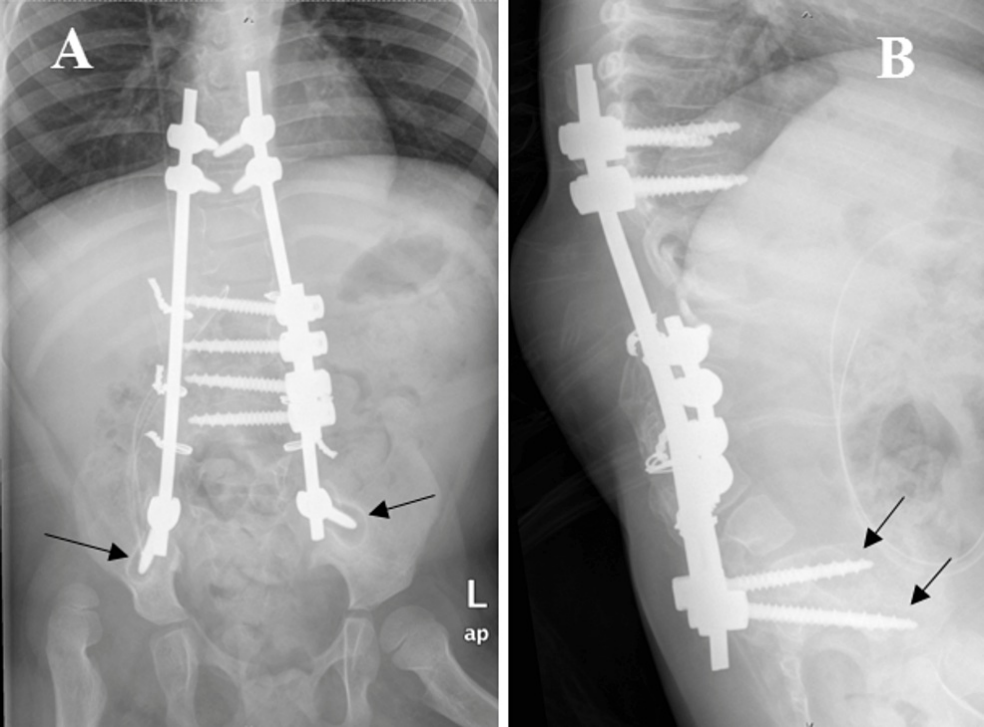
Whole spine x-ray anteroposterior view (A) and lateral view (B) during four years follow-up showing the implant in situ with maintained thoracolumbar curvature and union achieved. The x-ray also showed minimal aseptic loosening of bilateral iliac screws (arrows) which was asymptomatic clinically and was treated conservatively.

At four years of follow-up in our spine clinic, there is no loss of deformity correction and revealed good surgical outcomes (Figure 7, panel A and B). We noted there is minimal aseptic loosening of bilateral iliac screws throughout our follow-up for the past four years and we treated the aseptic loosening of screws conservatively in view of the patient being asymptomatic clinically (Figure 7, panel A and B). The surgical scar showed good healing with no more ulceration over the back (Figure 4, panel B). No revision surgery is needed and no rod migration nor deterioration of the kyphoscoliotic curvature was noted for the past four years.

## Discussion

Sharrard was the first who described kyphectomy for the treatment of myelomeningocele-related severe kyphosis in 1968 [5]. Multiple methods of fixation and correction following kyphectomy for achieving spondylodesis in kyphectomy have been reported, including pedicle screw instrumentation, Harrington rods, wiring, cables, hooks, and anterior and posterior plating. Corrective procedures for congenital kyphotic deformity in patients with myelomeningocele are surgically challenging and the complication rates are high. Skin breakdown, wound healing, prominent instrumentation, and loss of corrective curvature are the complications that are commonly encountered [6,7].

The corrective surgery aims to achieve a balanced, stable, and fused spine that allows free mobilization, and balanced sitting, subsequently restoring a good sagittal balance and minimizing the risk of skin ulceration over a gibbus [8]. Sitting balance without arm supports is important, especially in a non-ambulatory group of patients.

A single posterior spinal instrumentation alone is difficult to prevent the progression of spinal kyphoscoliosis. Winter et al. reported that the management with posterior fusion alone in cases with kyphotic curvature 50° or more resulted in a 64% incidence of failure. Winter et al. also reported a better surgical outcome in a two-stage of combined posterior and anterior spinal instrumentation, where stabilization was achieved with the osseous union of 80% (16 out of 20 patients) versus 20% of non-union (four out of 20 patients) [9].

There are additional advantages to a single-stage surgery which are reducing in risks of repositioning, improving the total operative duration, and shortening the overall duration of hospitalization and potential morbidities. Overall usage of anesthesia drugs on patients will be minimized as well [10].

In the present case, the spinal cord was exposed followed by dissection and release of the fibrous tissue adhering to the undersurface of the dura. A total kyphectomy of L3 vertebrae was performed to shorten the spinal column to prevent the spinal cord from over-distraction. Kyphectomy also allowed a better skin closure where initially the skin was impinged by the protruded segment of the kyphotic deformity. The recurrence of ulceration over the apex of the kyphosis was also been reduced. Spinal instrumentations were then applied anteriorly from L1 to L5 and posteriorly from T10 to the pelvis. Somatosensory evoked potential (SSEP) monitoring was utilized during the surgery.

There is a risk of deterioration or worsening of kyphotic curvature and recurrence of ulceration over the kyphosis along the patient’s growth. Therefore, close and careful monitoring of the patient post-operatively is crucial. Proper monitoring post-operatively enables us to ensure the instrumentation implants are in situ, the alignment of the fixation is well maintained, and the surgical scar is completely healed without recurrence ulceration upon the follow-up in the clinic.

The kyphoscoliotic deformities in the present case were corrected and solid fusion was achieved. The spine curvature was well maintained with minimal aseptic loosening of bilateral iliac screws noted during the follow-up. The aseptic loosening of the screws was treated conservatively in view of the patient being asymptomatic clinically. The patient is free from any wound or metalwork-related infections upon four years follow-up in the clinic.

The simultaneous anterior and posterior instrumentation together with total kyphectomy in a single-stage kyphoscoliotic corrective surgery is challenging and technically demanding. A meticulous surgical technique and proper handling of patients will result in meritorious surgical outcomes for patients with kyphoscoliosis deformity.

## Conclusions

The kyphotic deformity with refractory ulceration is a major issue in patients with myelomeningocele. The anterior and posterior spinal instrumentation combined with kyphectomy in a single-stage surgery is a successful surgical strategy in managing patients with severe kyphoscoliosis in myelomeningocele. An early and appropriate pre-operative planning for deformity corrective surgery is the key to success in order to achieve satisfactory deformity curvature correction and reduce the recurrence of ulceration over the kyphotic curve with excellent long-term surgical outcomes.
